# Spontaneous Coronary Artery Dissection Presenting as Electrical Storm: A Case Report

**DOI:** 10.5811/cpcem.1372

**Published:** 2024-01-23

**Authors:** Nathan Escorial, Coleman Cowart, Matthew Paparian, Joel Stillings

**Affiliations:** Desert Regional Medical Center, Department of Emergency Medicine, Palm Springs, California

**Keywords:** *spontaneous coronary artery dissection*, *electrical storm*, *case report*

## Abstract

**Introduction:**

Spontaneous coronary artery dissection (SCAD) is an important cause of myocardial infarction in patients of younger age without typical atherosclerotic risk factors and can present with ventricular arrhythmia such as ventricular tachycardia (VT) or ventricular fibrillation (VF). Electrical storm (ES) is defined as greater than or equal to 3 episodes of VT or VF occurring within 24 hours.

**Case Report:**

A healthy 38-year-old-male developed chest pain while exercising at the gym and presented to the emergency department unresponsive in a ventricular arrhythmia defined as ES. The patient’s cardiac arrest was initially refractory to standard defibrillation and Advanced Cardiac Life Support medications. He was ultimately diagnosed with SCAD of the left anterior descending artery, which was stented. The patient survived neurologically intact after a 13-day hospital stay.

**Conclusion:**

Spontaneous coronary artery dissection is a significant cause of acute coronary syndrome and occurs in healthier patients without cardiac risk factors. Electrical storm represents a unique manifestation of SCAD. Emergency physicians should maintain a heightened suspicion for SCAD for accurate diagnosis and treatment.

Population Health Research Capsule What do we already know about this clinical entity?

*Spontaneous coronary artery dissection (SCAD) is the most common cause of pregnancy-associated myocardial infarction.*

What makes this presentation of disease reportable?

*We describe the case of a young male patient without any traditional atherosclerotic risk factors uniquely presenting with electrical storm.*

What is the major learning point?

*In younger patients with chest pain who present to the emergency department, SCAD should be included in the differential.*

How might this improve emergency medicine practice?

*Awareness of this clinical entity in younger patients, especially pregnant women, will facilitate diagnosis and management of acute coronary syndrome.*

## INTRODUCTION

Spontaneous coronary artery dissection (SCAD) is a significant cause of acute coronary syndrome (ACS), myocardial infarction (MI), and sudden death; it is particularly associated with female gender, pregnancy, and physical and emotional triggers.^1^ It may be the cause of up to 1–4% of ACS cases overall and up to 35% of MIs in women >50 years old; SCAD is the most common cause of pregnancy-associated MI.^1^ Spontaneous coronary artery dissection causes myocardial injury by causing an intramural hematoma resulting in coronary artery obstruction. Spontaneous coronary artery dissection is frequently misdiagnosed or underdiagnosed and treated like typical ACS.^2^

Electrical storm (ES) is a life-threatening syndrome involving recurrent episodes of ventricular arrhythmias, specifically ≥3 in a 24-hour period.^3^ Initial management is aimed at identifying the underlying cause, which is often myocardial ischemia. The most effective agents in terminating ES are β-blockers and amiodarone.^3^ It is estimated that ventricular arrhythmias occur in 8–14% of patients with SCAD.^2^ In the following report, we present a young male who presented in ES and was ultimately diagnosed with SCAD. Recognizing ES is paramount to correctly treating this diagnosis. If epinephrine is administered, per the standard used in Advanced Cardiac Life Support (ACLS), it may make these patients more prone to recurrent arrhythmias.^3^

## CASE REPORT

A 38-year-old male with no known past medical history presented to the hospital as a transfer from an outside facility status post cardiac arrest. The patient had been experiencing chest pain for two weeks prior to the arrest. On the day of the arrest, the patient had ingested a pre-workout nutritional supplement before working out at the gym. He then developed chest pain, and emergency medical services were called. Initially he had a normal electrocardiogram (ECG) and stable vital signs. Paramedics administered one dose of nitroglycerin en route to the emergency department (ED) at the first hospital. On arrival, he went into ventricular tachycardia (VT) and became unresponsive.

Cardiopulmonary resuscitation (CPR) was initiated, and he received one synchronized cardioversion of 150 joules (J) and a second shock of 200 J. He was intubated while CPR was in progress and had an emergent central line placed. During the initial arrest, the patient received in total 10 doses of one milligram (mg) epinephrine (1:10,000 dilution) intravenously (IV), three doses of 8.4% sodium bicarbonate 50 milliequivalent (mEq) IV, 300 mg amiodarone IV, two grams (g) of calcium gluconate IV, and 50 mg esmolol IV. During resuscitation the patient was defibrillated a total of six times, including the two initial defibrillations at 150 J and then 200 J, followed by four dual sequence defibrillations at 400 J, total for each attempt.

After 44 minutes of CPR, return of spontaneous circulation (ROSC) was achieved with a sinus bradycardia rhythm. However, the patient went into a second cardiac arrest, secondary to ventricular fibrillation (VF), with an additional three rounds of CPR before ROSC was achieved. The ECG after the second ROSC showed ST-segment elevations in V4, V5, V6, I and aVL with reciprocal changes in II, III and aVF. (ECG image unavailable) The patient then had a third cardiac arrest—a VT arrest—for which he was cardioverted and received one dose of epinephrine 1 mg IV and magnesium sulfate 2 g IV before ROSC was achieved.

We were unable to learn the exact times at which the arrests occurred or the length of the time between the arrests,as these had not been documented in the chart. We know the first arrest occurred at 2 pm; however, the details of the timing of each subsequent arrest were unclear. Other medications given at the first hospital included lidocaine 100 mg IV, propranolol 5 mg IV, aspirin 300 mg per rectum, 0.9% normal saline two liters (L) IV, post-intubation sedation with fentanyl and midazolam drips (guttae [gtt]). The patient was started on amiodarone gtt, lidocaine gtt, norepinephrine gtt, and sodium bicarbonate gtt. Laboratory values obtained from the ED visit are noted in Table 1. Of note, the patient had a normal sensitive troponin of greater than 800 nanograms per milliliter (ng/mL) (reference range: 0.0–0.08 ng/mL) consistent with myocardial infarction secondary to SCAD.

**Table 1.** Laboratory values obtained in the emergency department from a 38-year-old patient status post cardiac arrest.

Labs	Value	Reference range
1^st^ Troponin	0.61	0.0–0.08 ng/mL
2^nd^ Troponin	800	0.0–0.08 ng/mL
White blood cells	14.51	4.8–10.8 × 10^3^/mcl
Hemoglobin	13.6	14%–18%
Platelets	218 × 10^3^	130–400 × 10^3^/mcl
Sodium	143	136–145 mEq/L
Potassium	3.9	3.5–5.1 mEq/L
Carbon dioxide	15	21–32 mEq/L
Anion gap	23	4–14
Calcium	11.1	8.5–10.1 mg/dL
Blood urea nitrogen	17	7–18 mg/dL
Creatinine	1.9	0.6–1.3 mg/dL
Glomerular filtration rate	40	normal high < 60
Glucose	324	74–106 mg/dL
1^st^ Alanine transaminase	310	30–65 IU/L
2^nd^ Alanine transaminase	2372	30–65 IU/L
1^st^ Aspartate transaminase	254	15–37 IU/L
2^nd^ Aspartate transaminase	842	15–37 IU/L
Lipase	274	73–393 IU/L
Arterial blood gas		
pH	6.854	7.35–7.45
Carbon dioxide	34.2	35–45 mm Hg
Oxygen	144.2	60–80 mm Hg
Bicarbonate	5.9	20–26 mm Hg
Base excess	−27.5	−3 to 3 mmol/L

*ng/mL*, nanograms per milliliter; *mcL*, microliters; *mEq/L*, milliequivalents per liter; *IU*, international units; *mm HG*, millimeters of mercury; mmol, millimole.

**Image 1.**
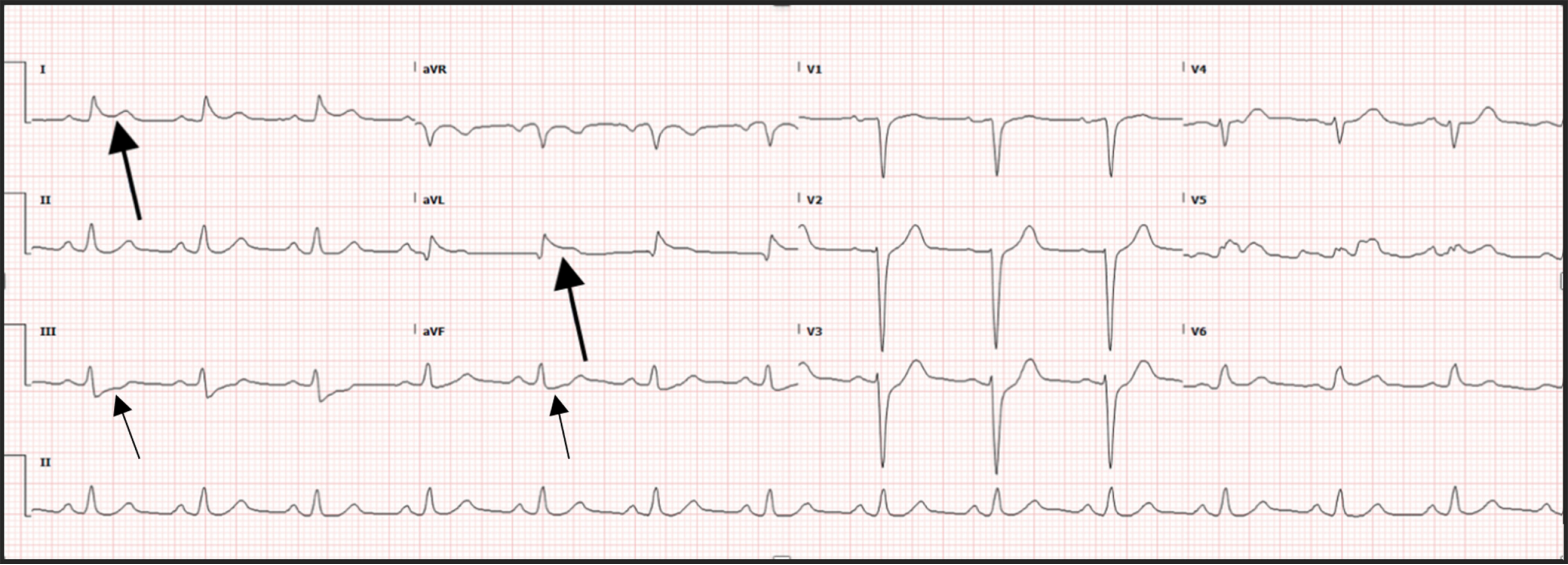
Electrocardiogram in a patient status post cardiac arrest demonstrating ST-segment elevations in lead I and aVL (large arrows). There are also ST-segment depressions in leads III and aVF (small arrows).

**Image 2.**
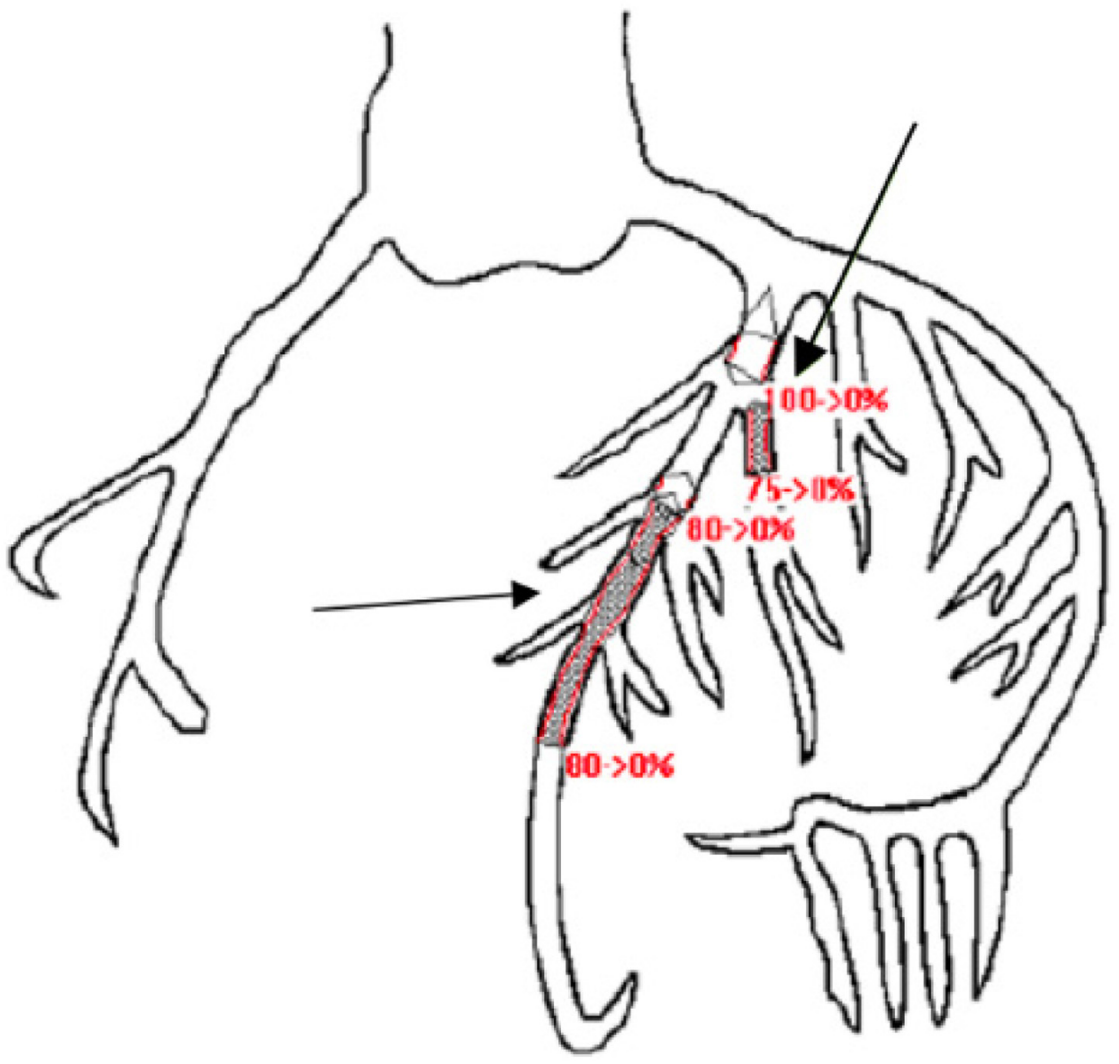
Coronary artery tree from cath lab report in a patient status post cardiac arrest. Shaded areas represent areas of occlusion. Red percentage values indicate the amount of occlusion prior to stenting. Lower arrow indicates the proximal to mid-left anterior descending artery. Upper arrow indicates the proximal diagonal branch off the left anterior descending artery. Shaded areas were stented.

The patient was transferred to the regional trauma center for interventional cardiology for the diagnosis of acute ST-segment elevation myocardial infarction (STEMI), ES, status post cardiac arrest. Upon arrival to the higher level of care facility, a repeat ECG showed ST-segment elevation in I and aVL with depressions in III and aVF (Image 1). The patient was taken to the catheterization lab for percutaneous coronary intervention (PCI) and was diagnosed with SCAD resulting in acute total occlusion of the left anterior descending (LAD) artery at its proximal segment with an extension of intramural hematoma into the long segment of the mid LAD, as well as into the proximal major diagonal branch (Image 2). The patient had three overlapping drug-eluting stents placed in the proximal to mid LAD and one stent placed in the proximal major diagonal branch off the LAD.

The patient was diagnosed with severe dilated cardiomyopathy with an ejection fraction of 15% and severe hypokinesis in all mid and apical left ventricular segments and stress-induced cardiomyopathy. One week later the patient had an internal cardiac defibrillator placed and was discharged to acute rehabilitation after a 13-day hospital stay. He survived neurologically intact. After acute rehabilitation and discharge, the patient subsequently presented to the ED frequently and required hospital admissions for complications related to his SCAD. Specifically, he suffered from consequences related to heart failure exacerbations, requires supplemental oxygen at home, and takes apixaban for atrial fibrillation.

## DISCUSSION

This patient presented to the hospital in cardiac arrest. He met criteria for ES with multiple rounds of ACLS with defibrillation and cardioversion including dual sequence, despite IV amiodarone, esmolol and propranolol, which are the recommended antiarrhythmics for terminating ES.^4^ It is thought that most patients with ES have severe underlying structural heart disease or other known triggers such as drug toxicity, electrolyte disturbances, acute myocardial ischemia, thyrotoxicosis, or QT prolongation.^4^ In this case, the patient presented with STEMI with elevated cardiac biomarkers and ES secondary to SCAD of his LAD and proximal diagonal branch. Patients who survive and present for initial evaluation of SCAD almost universally experience ACS and elevated troponin.^1^ The gold standard of diagnosis for SCAD is coronary angiography.^1^ Treatment varies between conservative therapy with inpatient monitoring vs coronary artery bypass grafting vs PCI.

Our patient was taken for PCI due to his hemodynamic instability and active ischemia. Interestingly, the patient had ingested a pre-workout supplement known to contain multiple ingredients such as nitric oxide, caffeine, beta-alanine, and nitric oxide agents, with limited data on safety.^5^ The patient was undergoing physical exertion at the gym, which is the most commonly reported trigger and precipitating factor in 28.9% of cases of SCAD.^6^ He had been experiencing chest pain for two weeks prior to his presentation to the hospital. This highlights the fact that there are varying presentations of SCAD ranging from mild chest pain to sudden cardiac death.^3^ Given SCAD’s underdiagnosis and unknown prevalence, it is difficult to state whether this patient’s presentation was typical as there are limited case reports of young men with SCAD. He does not fit the typical risk profile of patients previously diagnosed with SCAD who are typically healthy, young and middle-aged individuals, particularly women, without traditional cardiovascular risk factors.^1^ While ventricular arrhythmias or sudden cardiac death are reportedly present in up to 3–11% in patients with SCAD, our patient presented in ES. There are limited reports on the prevalence of ES as the clinical presentation of SCAD.

## CONCLUSION

It seems likely that the combination of physical exertion, unknown underlying genetic risk factors, and unknown family history had a cumulative effect in precipitating SCAD and, therefore, electrical storm. We do not know the specific pre-workout supplement the patient ingested, its ingredients, or whether it contained stimulants or had any effect at all regarding this patient’s presentation. It remains an interesting question as to another trigger of this patient’s SCAD. The recommended treatment for ES in patients in cardiac arrest includes adrenergic blockade with propranolol as the first-line agent. which is not typically used in the ACLS algorithm.^7^ The use of epinephrine may make the patient pro-arrhythmic, further perpetuating ventricular arrhythmias and ES; therefore, it should not be used as a standard part of ACLS in this clinical scenario, highlighting the importance for emergency physicians of recognizing electrical storm early.^3^

This patient was treated with a multitude of cardioactive medications, electricity, and ultimately PCI and was eventually discharged from the hospital neurologically intact. Despite the increased recognition of SCAD, there are large gaps in knowledge regarding this disease entity as a clinical presentation for acute coronary syndrome. If SCAD is suspected, emergency physicians should consult with cardiology early, as coronary angiography should be performed as soon as possible. Physicians must maintain a heightened suspicion for SCAD as a potential diagnosis for a wide array of presentations related to ACS.
